# Picropodophyllotoxin Induces G1 Cell Cycle Arrest and Apoptosis in Human Colorectal Cancer Cells via ROS Generation and Activation of p38 MAPK Signaling Pathway

**DOI:** 10.4014/jmb.2109.09012

**Published:** 2021-09-15

**Authors:** Seung-On Lee, Ah-Won Kwak, Mee-Hyun Lee, Ji-Hye Seo, Seung-Sik Cho, Goo Yoon, Jung-Il Chae, Sang Hoon Joo, Jung-Hyun Shim

**Affiliations:** 1 Department of Biomedicine, Health and Life Convergence Sciences, BK21 Four, Biomedical and Healthcare Research Institute, Mokpo National University, Muan, Jeonnam 58554, Republic of Korea; 2 Department of Pharmacy, College of Pharmacy, Mokpo National University, Muan, Jeonnam 58554, Republic of Korea; 3 College of Korean Medicine, Dongshin University, Naju, Jeonnam 58245, Republic of Korea; 4 Department of Dental Pharmacology, School of Dentistry, Jeonbuk National University, Jeonju, Jeonbuk 54896, Republic of Korea; 5 College of Pharmacy, Daegu Catholic University, Gyeongsan, Gyeongbuk 38430, Republic of Korea; 6 The China-US (Henan) Hormel Cancer Institute, Zhengzhou, Henan, 450008, P.R. China

**Keywords:** Picropodophyllotoxin, colon cancer, cell cycle arrest, reactive oxygen species, p38, apoptosis

## Abstract

Picropodophyllotoxin (PPT), an epimer of podophyllotoxin, is derived from the roots of *Podophyllum hexandrum* and exerts various biological effects, including anti-proliferation activity. However, the effect of PPT on colorectal cancer cells and the associated cellular mechanisms have not been studied. In the present study, we explored the anticancer activity of PPT and its underlying mechanisms in HCT116 cells. The 3-(4,5-dimethylthiazol-2-yl)-2,5-diphenyltetrazolium bromide (MTT) assay was used to monitor cell viability. Flow cytometry was used to evaluate cell cycle distribution, the induction of apoptosis, the level of reactive oxygen species (ROS), assess the mitochondrial membrane potential (Δψm), and multi-caspase activity. Western blot assays were performed to detect the expression of cell cycle regulatory proteins, apoptosis-related proteins, and p38 MAPK (mitogen-activated protein kinase). We found that PPT induced apoptosis, cell cycle arrest at the G1 phase, and ROS in the HCT116 cell line. In addition, PPT enhanced the phosphorylation of p38 MAPK, which regulates apoptosis and PPT-induced apoptosis. The phosphorylation of p38 MAPK was inhibited by an antioxidant agent (*N*-acetyl-L-cysteine, NAC) and a p38 inhibitor (SB203580). PPT induced depolarization of the mitochondrial inner membrane and caspase-dependent apoptosis, which was attenuated by exposure to Z-VAD-FMK. Overall, these data indicate that PPT induced G1 arrest and apoptosis via ROS generation and activation of the p38 MAPK signaling pathway.

## Introduction

Colorectal cancer is predicted to be the third most commonly diagnosed cancer and the second leading cause of cancer death worldwide, with about 1.93 million new cases and 935,000 deaths worldwide in 2020 [1]. Despite recent improvements in cancer treatment, the survival rate of colorectal cancer patients is relatively low at around 60% [2]. Surgery, adjuvant radiotherapy, and chemotherapy are the main colorectal cancer treatment [3]. Until now, 5-fluorouracil has been the first line chemotherapy against colorectal cancer [4]. Although 5-fluorouracil based treatment has yielded an improvement in overall survival, a large number of patients with colorectal cancer experience relapse after 5-fluorouracil-based chemotherapy due to acquired resistance [5]. The clinical application of 5-fluorouracil has also been limited by systemic adverse effects such as gastrointestinal reactions, hair loss, bone marrow suppression, and decreased white blood cells and platelets [6].

Phytochemicals from various natural sources such as plants, insects, animals, and microorganisms have recently received attention as a novel therapeutic approach to cancer treatment due to lower cost and fewer side effects [7]. Podophyllotoxin, derived from *Podophyllum* species, exerts strong anti cancer activity against various cancer types by inhibiting microtubule assembly [8]. However, the application of podophyllotoxin as a chemotherapeutic agent in clinical therapy has been limited due to its high toxicity and low bioavailability [9]. Picropodophyllotoxin (PPT) is an epimer of podophyllotoxin, which modulates tubulin polymerization and the insulin-like growth factor I receptor with enhanced selectivity for cancer cells and reduced systemic toxicity [10]. A previous study reported the anti-proliferative effects of PPT in human esophageal squamous cell carcinoma cells [8].

A moderate increase in intracellular reactive oxygen species (ROS) in normal cells can promote cell proliferation and differentiation, whereas excessive ROS production causes oxidative damage to biological molecules including lipids, proteins, and DNA, and results in apoptosis and cell death [11, 12]. Because they have a higher basal ROS content than normal cells, cancer cells are more susceptible to acute increase in intracellular ROS generation [13]. Therefore, phytochemicals elevating cellular ROS production can be exploited as effective chemotherapeutic agents to cancer cells preferentially and selectively [14].

ROS have been related to the activation of the mitogen-activated protein kinase signaling pathway [15]. C-Jun N-terminal kinase (JNK) and p38, belonging to the mitogen-activated protein kinase (MAPK) family, are activated by various stresses including heat, osmotic shock, UV irradiation, and oxidative stress [16]. The stress-activated JNK and p38 pathways play a critical role in cell cycle arrest, cell growth inhibition, and apoptotic cell death regulation [16]. Apoptosis, programmed cell death, is driven by two signaling pathways known as the extrinsic and intrinsic pathways [17]. The extrinsic pathway initiates apoptosis by the binding of extracellular ligands to death receptors, which, in turn, activates the caspase cascade [17]. Activated caspase-8 cleaves the cytosolic BH3-only protein Bid to produce a truncated form of Bid (t-Bid), which then bridges from the extrinsic to the intrinsic pathway by decreasing the mitochondrial membrane potential (MMP) [18]. The intrinsic pathway is mediated by the opening of permeability transition pores, allowing the release of cytochrome c from mitochondria into the cytosol, which forms a complex with apoptotic protease-activating factor 1 (Apaf-1) and caspase-9 [17]. Active caspase-9 activates downstream executioner caspases, resulting in DNA fragmentation and apoptosis [19]. The anti- and pro-apoptotic Bcl-2 family proteins are major regulators of apoptosis, and the activity of Bcl-2 family proteins is affected by JNK and p38 activation [20, 21].

The aim of this study was to examine the effect of PPT on the proliferation and apoptosis of colorectal cancer cell, and investigate the novel mechanism of the anticancer effect. We explored the mechanism of the anti cancer effect of PPT by analyzing its targets including ROS, the MAPK signaling pathway, ER-stress, and the apoptosis pathway.

## Materials and Methods

### Materials

Roswell Park Memorial Institute (RPMI)-1640 medium was purchased from Gibco (UK). Antibodies against actin, p21, p27, C/EBP homologous protein (CHOP), death receptor 4 (DR4), DR5, Bid, Bcl-xl, Bad, Mcl-1, Apaf-1, 78-kDa glucose regulated protein (GRP78), cyclin D1, CDK6, CDK2 and caspase-3 were purchased from Santa Cruz Biotechnology, Inc. (USA). Antibodies against p38 and phospho (p)-p38 (Thr180/Try182) were obtained from Cell Signaling Technology (USA). Basal Medium Eagle, carbobenzoxy-valyl-alanyl-aspartyl-(O-methyl)-fluoromethylketone (Z-VAD-FMK), dimethyl sulfoxide (DMSO), N-acetyl-L-cysteine (NAC), 3-(4,5-dimethylthiazol-2-yl)-2,5-diphenyl tetrazolium bromide (MTT), and p38 inhibitor (SB203580), picropodophyllotoxin (PPT), and Tween 20 were procured from Sigma-Aldrich (USA). Cell culture medium, fetal bovine serum (FBS), penicillin/streptomycin (p/s), and phosphate-buffered saline (PBS) were purchased from Hyclone (USA).

### Cell Culture and Treatment with PPT

Human colorectal cancer HCT116 cells, from the American Type Culture Collection (USA) were cultured in RPMI-1640 medium supplemented with 10% FBS and p/s (100 U/ml). The cells were incubated at 37°C in a humidified air with 5% CO_2_. The cells were treated with PPT dissolved in DMSO at varying concentrations (0, 0.1, 0.2, and 0.3 μM). The incubation time was 24 or 48 h unless stated otherwise.

### The 3-(4,5-Dimethylthiazol-2-yl)-2,5-Diphenyltetrazolium Bromide (MTT) Cell Viability Assay

HCT116 cells (5,000 cells in each well) were seeded in 96-well plate one day before PPT treatment. The cells were allowed to grow for 24 or 48 h after treatment. After incubation with PPT, MTT reagent was added (0.5 mg/ml final concentration) to each well, and incubated at 37°C for 1 h. DMSO (100 μl) was added to each well to dissolve the formazan crystals. The absorbance of the sample was measured at 570 nm using a Multiskan GO spectrophotometer (Thermo Scientific, Finland), and the relative cell viability was calculated compared to the negative control (0 μM PPT in DMSO).

### Soft Agar Colony Formation Assay

Soft agar growth was used to assess anchorage-independent growth. The bottom layer was prepared by pouring 0.6% agar (1 ml/in each well of a 6-well plate) containing culture medium and PPT dissolved in DMSO. After the bottom layer was solidified, HCT116 cells (8 × 10^3^ cells/well) were seeded with 0.3% agar in culture medium and PPT dissolved in DMSO. After incubation for a week, the colony numbers and sizes were measured using a light microscope (Leica Microsystems, Germany). The relative colony number and size were calculated by comparing them to the negative controls (DMSO only).

### Annexin V Apoptosis Assay with Annexin V/7-aminoactinomycin D (7-AAD) Double Staining

The Muse Annexin V & Dead Cell Kit from EMD Millipore (USA) was used to analyze the apoptosis of HCT116 cells. First, the cells were treated with PPT for 48 h before the annexin V apoptosis assay. After incubation, the cells were harvested and washed with 1 × PBS before staining with Muse Annexin V & Dead Cell Reagents. After further incubation for 20 min at room temperature (RT) in the dark, flow cytometry analysis was performed using a Muse Cell Analyzer (EMD Millipore). In the flow cytometry density plot, the cells were sorted into live (bottom left side, annexin V negative/7-AAD negative), early apoptotic (bottom right side, annexin V positive/7-AAD negative), late apoptotic (top right side, annexin V positive/7-AAD positive), and necrotic (top left side, annexin V negative/7-AAD positive) cells. The total number of apoptotic cells was calculated by adding the early and late apoptotic cells.

### Cell Cycle Analysis

To monitor the cell cycle distribution of HCT116 cells treated with PPT, the Muse Cell Cycle Kit from EMD Millipore was used according to the manufacturer’s manual. Briefly, cells treated with PPT were harvested and washed with ice-cold 1 × PBS. Then 70% ethanol was added for overnight fixing at -20°C. The fixed cells were collected and rinsed with 1 × PBS. Muse Cell Cycle Reagent was added for 30 min of incubation at RT in the dark. Flow cytometry analysis was performed using a Muse Cell Analyzer to analyze the DNA content.

### Intracellular ROS Measurements

The intracellular ROS levels of HCT116 cells treated with PPT were measured using a Muse Oxidative Stress Kit from EMD Millipore following the vendor’s instruction. In brief, cells treated with PPT were harvested and washed with 1 × assay buffer before incubation with Muse Oxidative Stress Reagent working solution for 30 min at 37°C. A Muse Cell Analyzer was used to determine the intracellular ROS levels.

### MMP Measurement

The MMP of HCT116 cells treated with PPT was measured using a Muse MitoPotential Kit. First, cells treated with PPT were harvested and washed with 1 × assay buffer. The washed cells were resuspended in Muse MitoPotential working solution, then incubated for 20 min at 37°C. Muse MitoPotential 7-AAD Reagent was added for 5 min of incubation at RT before flow cytometry analysis using a Muse Cell Analyzer. The cells were sorted in the flow cytometry density plot into live cells with intact MMP (bottom right side, Mitopotential+/7-AAD-), depolarized live cells (bottom left side, Mitopotential-/7AAD-), dead cells (top right side, Mitopotential+/7AAD+), depolarized dead cells (top left side, Mitopotential-/7AAD+).

### Multi-Caspase Activity Assay

Multi-caspase activity (caspase-1, -3, -4, -5, -6, -7, -8 and -9) was assessed with a Muse Multi-caspase Kit from EMD Millipore as directed by the manufacturer. Briefly, HCT116 cells treated with PPT for 48 h were harvested and washed with 1 × caspase buffer. After washing, the cells were resuspended in Muse Multi-caspase Reagent working solution for 30 min of incubation at 37°C. Then, Muse Caspase 7-AAD working solution was added for 5 min of incubation. Multi-caspase activity was determined using a Muse Cell Analyzer.

### Western Blot Assay

To lyse the cells, the harvested cells were washed with cold 1 × PBS, and then resuspended for sonication in RIPA buffer (iNtRON Biotechnology, Korea). Equal amounts of protein samples were resolved by 6−15% sodium dodecyl sulfate polyacrylamide gel electrophoresis after determining the protein concentration of the cell lysates using a Bio-Rad DC Protein Assay kit (USA), The resolved proteins were transferred to poly vinylidene fluoride membranes (EMD Millipore). Three percent or 5% skim milk in 1 × PBST (PBS with 0.1% Tween 20) was used to block the membranes by incubation for 2 h at RT. The respective primary antibodies were used to probe the membranes. The diluted primary antibodies (1:1,000 dilution in 1 × PBST) were incubated with the membranes either 2 h at RT or overnight at 4°C. Next, the membranes were washed with PBST, then probed with the secondary antibodies conjugated with horseradish peroxidase (1:4,000 – 6,000 dilution) by incubation for 1 h at RT. Visualization of the Western blots was done using Santa Cruz Western blotting luminol reagent (USA), and ImageQuant LAS 500 from GE Healthcare (Sweden) was used to detect the images.

### Statistical Analysis

The data are presented as the mean ± standard deviation (SD) when applicable, based on three independent triplicate experiments. The significance of the variation among multiple groups was assessed using one-way ANOVA. The Student’s *t*-test was used to compare two means. *p* values of less than 0.05 (*), 0.01 (**), and 0.001 (***) were considered statistically significant.

## Results

### PPT Suppresses Cell Proliferation and Induces Apoptosis in HCT116 Cells.

**Fig. 1:**
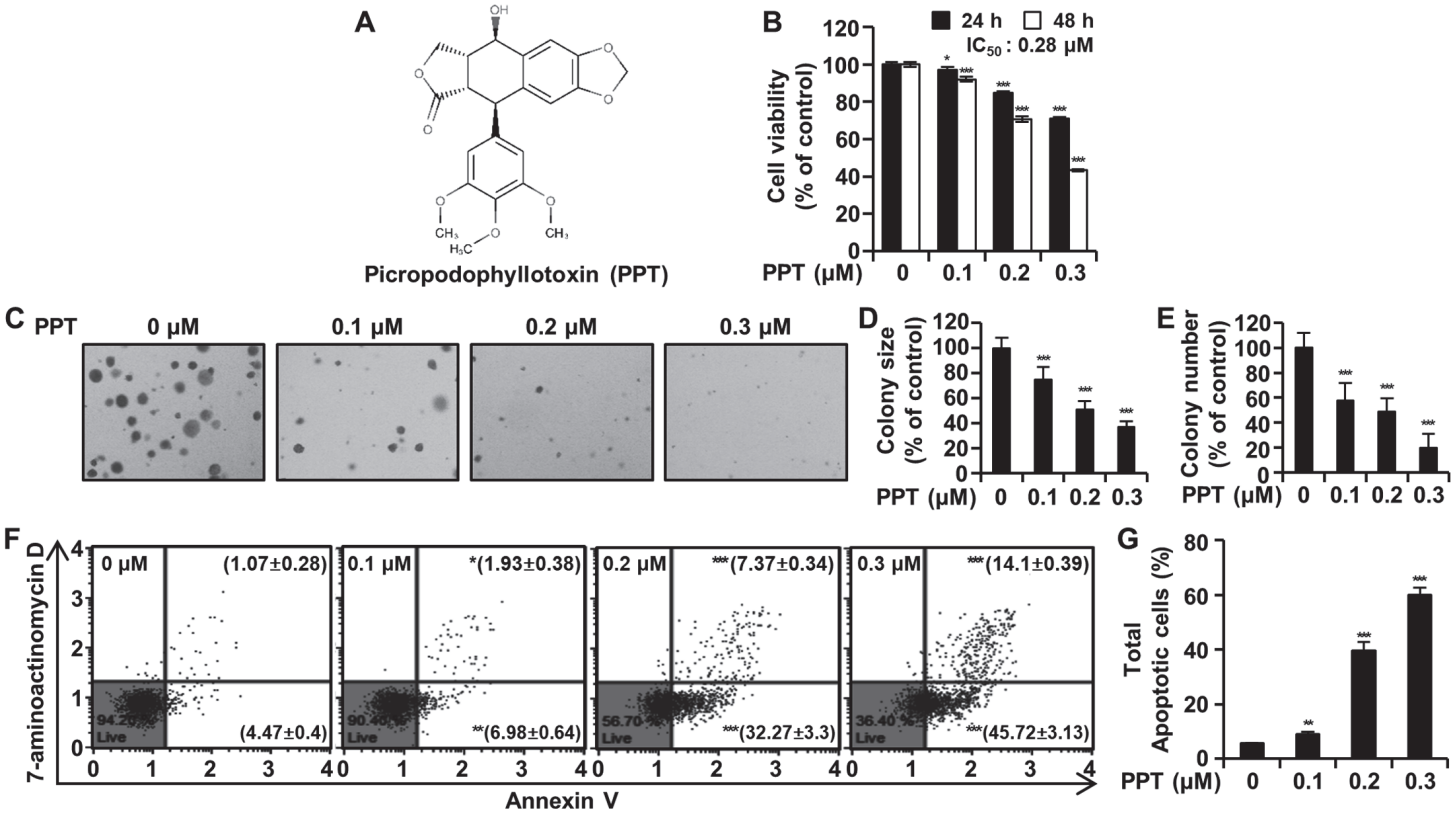
Effects of PPT on Cell Proliferation and Apoptosis in HCT116 Cells. (**A**) Chemical structure of picropodophyllotoxin. (**B**) HCT116 cells were treated with various concentrations of PPT for 24 and 48 h. Cell viability was detected by the MTT assay. (**C-E**) HCT116 cells were plated in soft agar containing DMSO (control) and PPT (0.1, 0.2, and 0.3 μM) for 7 days. Colony number and size were analyzed and expressed relative to the DMSO-treated control value. (**F** and **G**) HCT116 cells were incubated with the indicated concentrations of PPT for 48 h and analyzed by the Muse cell Analyzer using the Muse Annexin V & Dead Cell Kit. Live cells (annexin-V negative/7-AAD negative) are on the bottom left side, early apoptotic cells (annexin-V positive/7-AAD negative) on the bottom right side, late apoptotic cells (annexin-V positive/7-AAD positive) on the top right side and necrotic cells (annexin-V negative/7-AAD positive) on the top left side. The data are expressed as the mean ± SD (*n* = 3). *p < 0.05, **p < 0.01, and ***p < 0.001 compared to the control group.

First, we determined if PPT (Fig. 1A) had an anti-proliferative effect on HCT 116 cells. The MTT cell viability assay showed a significant decrease in the viability of HCT116 cells treated with PPT in concentration- (0.1, 0.2, and 0.3 μM) and time- (24 or 48 h) dependent manners (Fig. 1B). The IC_50_ values of inhibition of HCT116 cell growth were 0.55 μM at 24 h and 0.28 μM at 48 h (Fig. 1B). A colony formation assay using soft agar was performed to study the long-term effects of PPT on the proliferation of HCT116 cells. Treatment of HCT116 cells with PPT gradually inhibited both colony numbers and sizes in concentration-dependent manners (Figs. 1C-E). The annexin V apoptosis assay with annexin V/7-AAD double staining was used to investigate whether PPT induced apoptosis in HCT116 cells. The percentage of total apoptotic cells following treatment with 0, 0.1, 0.2, and 0.3 μM PPT was 5.53 ± 0.26%, 8.92 ± 0.97%, 39.63 ± 3.00% and 59.82 ± 2.89% (Figs. 1F and 1G). Taken together, the anti-proliferative effect of PPT led to HCT116 cell apoptosis.

### PPT Triggers Cell Cycle Arrest in the G1 Phase and Affects Cell Cycle Regulatory Proteins

**Fig. 2:**
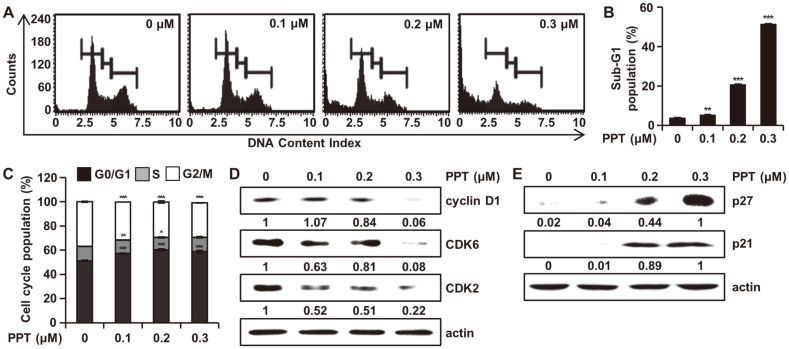
Effect of PPT on Cell Cycle Distribution in HCT116 Cells. (**A**, **B**, and **C**) HCT116 cells were treated with DMSO and PPT (0.1, 0.2, and 0.3 μM) for 48h. Cell cycle progression was assessed by the Muse Cell Analyzer. The mean ± SD of three independent measurements each with ratios from triplicates. *p < 0.05, **p < 0.01, and ***p < 0.001 compared to untreated group. (**D** and **E**) Cell lysates were prepared and analyzed by Western blotting for cyclin D1, CDK6, CDK2, p21, and p27. Actin was used as the denominator to quantify relative protein expression levels.

To investigate whether the inhibition of cell proliferation induced by PPT was related to cell cycle arrest, cell cycle analysis was performed using the Muse Cell Cycle Kit. Incubating HCT116 cells with different PPT concentrations for 48 h led to the dose-dependent arrest of HCT116 cells in the G1 phase of the cell cycle, which was accompanied by an increase in G1 phase cells (Fig. 2A). The sub-G1 phase cell cycle population was 3.80 ± 0.30%, 5.27 ± 0.45%, 20.73 ± 0.40% and 51.47 ± 0.40% at 0, 0.1, 0.2, and 0.3 μM concentrations of PPT, respectively (Fig. 2B). The percentage of cells distributed in the G1 phase in the 0.3 μM PPT-treated group was significantly higher than that in the untreated group (Fig. 2C). We assessed the protein levels of cyclin D1, CDK2, CDK6, p21, and p27 to elucidate the mechanisms of PPT-induced cell cycle arrest in HCT116 cells. The Western blot analysis revealed that the expression of cyclin D1, CDK2, and CDK6 was downregulated (Fig. 2D), and the expression of p21 and p27 was upregulated (Fig. 2E). These results indicated that G1 phase arrest may be associated with the anti-proliferative effect of PPT on HCT116 cells.

### PPT Causes ROS Accumulation and ER Stress in HCT116 Cells

**Fig. 3:**
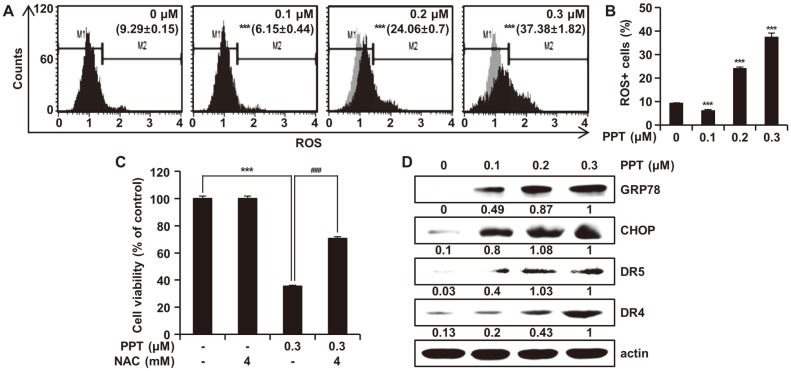
Effects of PPT on ROS Generation and ER Stress. (**A** and **B**) HCT116 cells were treated with 0, 0.1, 0.2, and 0.3 μM of PPT for 48 h and then analyzed for the level of cellular ROS using the Muse Oxidative Stress Kit. M1 represents cell population without ROS (ROS-) and M2 indicates the cell population with ROS (ROS+). (**C**) HCT116 cells were pretreated with NAC for 3 h and then treated with different concentrations of PPT for 48 h. Cell viability was measured by the MTT assay. The values are representative of the mean ±SD of three independent triplicate experiments. ***p < 0.001 compared to controls; ###*p* < 0.001 compared to PPT-treated cells. (**D**) HCT116 cells were treated with PPT at varying concentrations for 48 h, and the cell lysates were analyzed by Western blot for protein levels of GRP78, CHOP, DR5, and DR4. Actin was used as a loading control.

We explored whether the PPT-induced apoptosis of HCT116 cells was associated with the production of ROS. As shown in Fig. 3A, ROS generation was significantly increased in HCT116 cells treated with PPT in a dose-dependent manner (Fig. 3B). To determine the correlation between ROS accumulation and PPT-mediated apoptosis, we investigated the effect of the intracellular ROS scavenger NAC on PPT-induced apoptotic cell death in HCT116 cells. As shown in Fig. 3C, pretreatment of HCT116 cells with NAC (4 mM) gradually attenuated PPT-mediated apoptosis by 35.37%. These data suggest that intracellular ROS was the upstream stimulus for the initiation of apoptosis by PPT treatment in HCT116 cells. To examine the potential role of ER stress in the apoptosis of HCT116 cells, the expression of proteins involved in the ER stress response was assessed after treatment of HCT116 cells with PPT. As shown in figure 3D, treatment with PPT resulted in the up-regulation of GRP78 and CHOP in a concentration-dependent manner. We also found that the protein levels of death receptors DR4 and DR5 increased in HCT116 cells in response to PPT treatment in a dose-dependent fashion, suggesting that PPT induced the death receptor-mediated apoptosis. These overall results indicated that PPT upregulated intracellular ROS generation in HCT116 cells, leading to the induction of oxidative stress-mediated apoptosis.

### PPT Causes Activation of p38 MAPK in HCT116 Cells

**Fig. 4:**
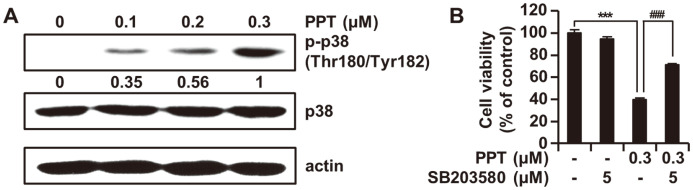
Effect of PPT on p38 MAPK Signaling Pathway. (**A**) HCT116 cells were stimulated with 0, 0.1, 0.2, and 0.3 μM of PPT for 48 h. Total cell lysates were collected and the protein levels of p38 and p-p38 were analyzed by Western blot. Actin was used as the internal control. (**B**) HCT116 cells were pre‐treated with 5 μM SB203580 for 3 h and then treated with or without 0.3 μM PPT for 48 h. Cell viability was analyzed by the MTT assay. The results are shown as the means ± SD of three independent experiments. ***p < 0.001 compared to the control group; ###*p* < 0.001 compared to the PPT-treated group.

We performed Western blot assay to examine the effects of PPT on p38 MAPK signaling and the role of these pathways in the PPT-induced apoptosis of HCT116 cells. PPT increased the levels of p-p38 in a concentration-dependent manner, whereas the levels of total p38 protein remained constant (Fig. 4A). To confirm if p38 played a role in the activation of the apoptotic pathway in PPT-treated HCT116 cells, cell viability, assayed with MTT in the presence of PPT or SB203580 was examined. The decrease in cell viability by PPT was reversed by p38 inhibitor (Fig. 4B). These results indicated that p38 phosphorylation induced by PPT was partly dependent upon the activation of p38 kinase.

### PPT-Induced Apoptosis is Mediated by Mitochondrial Pathways

**Fig. 5:**
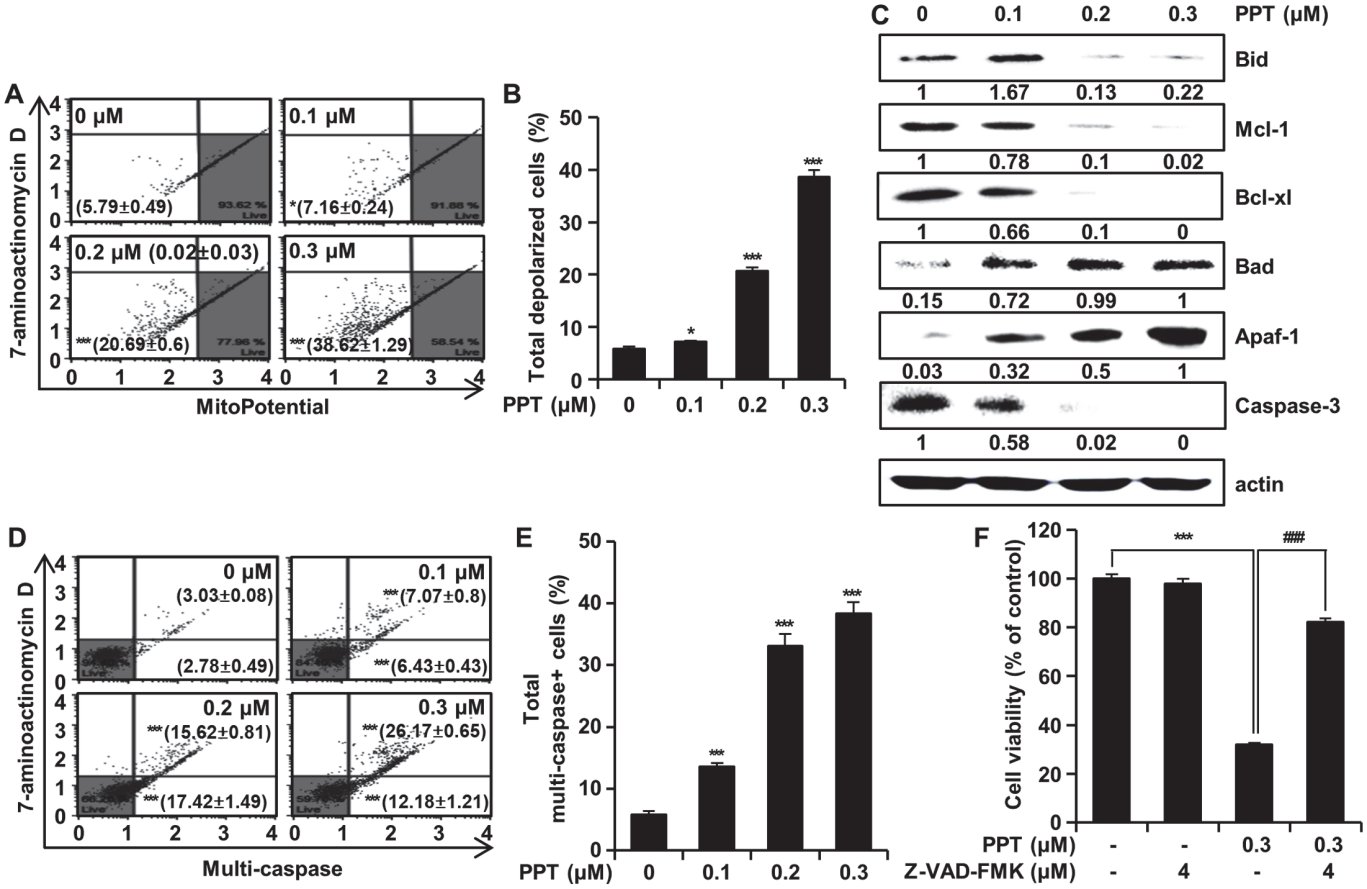
Effects of PPT on Mitochondrial-Mediated Apoptosis and Caspase Activation. (**A** and **B**) HCT116 cells were treated with PPT (0, 0.1, 0.2, and 0.3 μM) for 48 h and the extent of mitochondria membrane potential depolarization was measured using the Muse Mitopotential Kit. Combined MitoPotential dye and 7-AAD reactivity allowed classification of the cells into four groups, as follows: live cells with intact mitochondrial membrane potential (bottom right, Mitopotential+/7- AAD-), depolarized live cells (bottom left, Mitopotential-/ 7-AAD-), dead cells (top right, Mitopotential+/ 7-AAD+), and depolarized dead cells (top left, Mitopotential-/7-AAD+). (**C**) HCT116 cells were treated with PPT for 48h and then analyzed by Western blots with antibodies against Bad, Bid, Mcl-1, Bcl-xl, Apaf-1 and caspase-3. Actin was used as internal standard. (**D** and **E**) HCT116 cells were treated with PPT (0, 0.1, 0.2, and 0.3 μM) for 48 h and multi-caspase (caspase-1, -3, -4, -5, -6, -7, -8, and -9) was measured using the Muse MultiCaspase Kit. Cells on the bottom right side indicate caspase-positive/live cells whereas the cells on the top right side indicate caspase-positive/dead cells. (**F**) HCT116 cells were treated with a pan caspase inhibitor Z-VAD-FMK (4 μM) with or without PPT (0.3 μM) for 48h. Cell viability was measured by the MTT assay. All data shown represent the mean ± SD (*n* = 3). *p < 0.05 and ***p < 0.001 vs the control group; ###*p* < 0.001 vs the PPT-treated group.

The effect of PPT on the loss of the mitochondrial membranes was assessed after PPT treatment for 48 h. The percentage of depolarized cells increased from 5.79 ± 0.49% in the control group to 7.16 ± 0.24%, 20.71 ± 0.90%, and 38.62 ± 1.29% in the 0.1, 0.2, and 0.3 μM PPT-treated groups, respectively (Figs. 5A and 5B). To investigate the PPT-regulated mitochondrial apoptosis pathways, we analyzed the protein expression levels of Bcl-2 family members by Western blots. PPT significantly decreased the expression of Bid, Mcl-1, Bcl-xl as well as caspase-3 and increased the levels of Apaf-1 and Bad proteins (Fig. 5C). To examine whether PPT-induced apoptosis is through activation of the caspase pathway, we treated HCT116 cells with varying concentrations (0, 0.1, 0.2, and 0.3 μM) of PPT for 48 h, then performed a multi-caspase (caspase-1, -3, -4, -5, -6, -7, -8, and -9) assay using the Muse Cell Analyzer. The ratio of cells showing caspase activity increased significantly in a concentration-dependent manner (Figs. 5D and 5E). To confirm the role of caspase in PPT-induced apoptosis, the cells were exposed to PPT either alone or in combination with a pan caspase inhibitor (Z-VAD-FMK) for 48 h. The Z-VAD-FMK treatment significantly prevented the decrease in cell viability by PPT treatment (Fig. 5F).

## Discussion

Considering the poor survival rate and prognosis of patients with colorectal cancer due to the lack of efficacious therapy, it is imperative to further explore anticancer agents for colorectal cancer with fewer side effects and good efficiency [22]. In a previous study, we proved that PPT induced apoptosis in esophageal squamous cell carcinoma cells by activating JNK/p38 MAPK signaling [8]. However, the anticancer effect of PPT and the underlying molecular mechanisms on colorectal cancer cells has not yet been studied. In the present study, we comprehensively investigated the potential cytotoxic effects of PPT in colorectal cancer using the HCT116 cell line (Fig. 1). The cell viability of HCT116 cells assayed with MTT decreased indicating the inhibition of proliferation by PPT in a dose- and time-dependent manner. Colony formation also decreased upon treatment with PPT.

Apoptosis, a natural and programmed cell death process, can be a target of anticancer therapy by restoring the death process of cancer cells [23]. Conversely, cancer cells can proliferate by disabling the apoptotic pathway [24]. After confirming that PPT was cytotoxic to HCT116 cells, we determined if PPT induced apoptosis. The annexin V apoptosis assay showed that the rate of apoptosis increased significantly following PPT treatment.

To further elucidate the anti-proliferative effect induced by PPT, cell cycle distribution was analyzed in HCT116 cells. Upon treatment with PPT, cell cycle arrest in the G1 phase and increases in the sub-G1 cell population were observed in a dose-dependent manner (Figs. 2A and 2B). To further understand the molecular mechanism of cell cycle arrest induced by PPT, we turned our attention to cyclin D and CDKs. In the regulation of cell cycle progression, the G1 phase is one of the main check points [25], and cyclin D is involved in the progression of cells through the G1 phase [26]. Indeed, we observed a decrease in the level of cyclin D1 in response to PPT treatment in a dose-dependent manner (Fig. 2C). Moreover, a decrease in the levels of CDK2 and CDK6 accompanied the decrease in cyclin D1, suggesting the down regulation of the cyclin D1 and CDK complexes induced by PPT. In contrast, we found increases in the protein level of p21 and p27, inhibitors of CDKs (Fig. 2D). These results support the inhibition of cyclin D1/CDK2 and cyclin D1/CDK6 complexes by p21 and p27 following the treatment of HCT116 cells with PPT.

High levels of ROS play a key role in apoptosis by inducing ER stress and mitochondrial dysfunction [27-29]. ER is a central cellular organelle, and it is involved with various functions such as protein translocation, protein folding, and protein post-translational modification [30]. Recently, it was reported that apoptosis could be induced by ER stress in various types of cells [31]. We observed increased levels of intracellular ROS in PPT-treated cells compared to nontreated cells (Figs. 3A and 3B). Pretreatment of the cells with NAC, an inhibitor of ROS, attenuated the anti-proliferative activity of PPT (Fig. 3C), implying that high levels of ROS were an upstream regulator of PPT-induced apoptosis. To determine whether ER stress resulted from increased level of ROS after treatment of HCT116 cells with PPT, we analyzed the level of ER stress related proteins. The 78 kDa glucose-regulated protein (GRP78) and C/EBP homologous protein (CHOP) are well-known biomarkers of ER stress [32, 33], and the levels of these two proteins were elevated by PPT treatment (Fig. 3D). Moreover, we could could also found an increase in the levels of DR4 and DR5 proteins. We suspect these two proteins were upregulated by the transcription factor CHOP, possibly relaying ER stress to the extrinsic apoptotic pathway [34]. These results suggest that PPT induced apoptosis in colorectal cancer cells via an ROS-mediated ER stress pathway.

The MAPK signaling pathway can be activated in response to oxidative stress in the ER [35], and activated p38 MAPK induces either cell cycle arrest or apoptosis [36, 37]. In addition, the accumulation of ROS in the cell is associated with the phosphorylation of p38 MAPK [38]. In this study, PPT treatment increased the phosphorylation of p38 MAPK (Fig. 4A). Moreover, co-treatment with SB203580, an inhibitor of p38 MAPK, markedly reversed PPT-induced apoptosis in colon cancer cells, suggesting that the p38 MAPK pathway was an upstream signaling molecule involved in PPT-induced apoptosis.

The accumulation of ROS could result in the opening of mitochondrial membrane permeability pores [39]. We found that the MMP levels decreased in HCT116 cells treated with PPT (Figs. 5A and 5B). To investigate the involvement of the mitochondrial pathway in PPT-induced apoptosis, we monitored the protein levels of the Bcl-2 family members by Western blot. The Bcl-2 family members, comprised of proapoptotic (Bid, Bad, etc.) and antiapoptotic members (Bcl-xl, Mcl-1, etc.), are key regulators of apoptosis [40]. PPT treatment of HCT116 cells resulted in a concentration-dependent increase in the level of Bad whereas the level of Bid, Bcl-xL and Mcl-1 decreased (Fig. 5C), indicating a shift in the balance between proapoptotic and antiapoptotic Bcl-2 proteins [41]. Apaf-1 activates caspase-9, leading to the proteolytic cleavage of caspase-3, which is a key apoptotic executive caspase [42]. As expected, PPT increased the level of Apaf-1 and cleaved caspase-3 in a dose-dependent manner (Fig. 5C). In addition, we found that the activation of caspases, the central components of apoptosis [43], was increased by PPT treatment in colorectal cancer cells (Figs. 5D and 5E). Furthermore, pretreatment with Z-VAD-FMK (pan caspase inhibitor) resulted in a significant decrease in apoptosis (Fig. 5F), suggesting that PPT induced caspase-dependent apoptosis via the mitochondrial pathway.

This study investigated the apoptosis and cell cycle arrest induced by PPT in colorectal cancer HCT116 cells. Our study indicated that PPT induced G1 arrest and apoptosis via ROS generation and activation of the p38 MAPK signaling pathway.
